# Absence of force suppression in rabbit bladder correlates with low expression of heat shock protein 20

**DOI:** 10.1186/1472-6793-5-16

**Published:** 2005-11-02

**Authors:** Timothy W Batts, John S Walker, Richard A Murphy, Christopher M Rembold

**Affiliations:** 1 Cardiovascular Division, Department of Internal Medicine, University of Virginia, Charlottesville, Virginia 22908 USA; 2 Department of Molecular Physiology and Cellular Biophysics, University of Virginia, Charlottesville, Virginia 22908 USA

## Abstract

**Background:**

Nitroglycerin can induce relaxation of swine carotid artery without sustained reductions in [Ca^2+^]_i _or myosin regulatory light chain (MRLC) phosphorylation. This has been termed force suppression and been found to correlate with ser^16^-phosphorylation of heat shock protein 20 (HSP20). We tested for the existence of this mechanism in a smooth muscle that is not responsive to nitric oxide.

**Methods:**

Isometrically mounted mucosa free rabbit bladder strips were contracted with carbachol and relaxed with 8-Br-cGMP, forskolin, or isoprenaline.

**Results:**

Contraction was associated with a highly cooperative relation between MRLC phosphorylation and force such that very small increases in MRLC phosphorylation induced large increases in force. Relaxation induced by 8-Br-cGMP, forskolin, or isoprenaline did not shift the MRLC phosphorylation-force relation from that observed with carbachol alone, i.e. there was no force suppression. HSP20 content was negligible (approximately two hundred-fold less than swine carotid).

**Conclusion:**

The lack of force suppression in the absence of HSP20 is consistent with the hypothesized role for HSP20 in the force suppression observed in tonic smooth muscles.

## Background

Ca^2+^-dependent phosphorylation of the myosin regulatory light chain (MRLC) is accepted as the primary mechanism regulating contraction of smooth muscle in response to excitatory stimuli [1,2]. However, this myosin-linked phosphorylation mechanism does not fully explain all observations. Of particular physiological interest is relaxation induced in activated tissues by NO or NO-donors that increase [cGMP]. In both vascular smooth muscle [3] and corpus cavernosum [4,5], there are two mechanism for NO donors to reduce tone. 1) NO donors can reduce in myoplasmic [Ca^2+^], which decreases MRLC phosphorylation, a process termed "deactivation." Deactivation is demonstrated when a decline in force is associated with a dependence of force on MRLC phosphorylation similar to that observed with contractile agonists alone. 2) NO donors can also reduce force without reductions in myoplasmic [Ca^2+^] or MRLC phosphorylation, a mechanism termed "force suppression" [6]. Force suppression is demonstrated when a decline in force is associated with a rightward shift in the dependence of force on MRLC phosphorylation similar to that observed with contractile agonists alone [6]. We hypothesized that a thin filament mechanism, specifically ser^16 ^phosphorylation of heat shock protein was the mediator of force suppression [7].

The human umbilical artery was found to not express HSP20 and not exhibit NO dependent relaxation [8], suggesting a possible linkage between HSP20 and NO dependent relaxation. The rabbit bladder is a phasic urogenital smooth muscle that does not relax in response to NO. We therefore hypothesized that the rabbit bladder could be another test of the role of HSP20 in force suppression. We therefore tested 1) whether HSP20 is expressed in rabbit bladder and 2) whether force suppression occurs with relaxing agents in the rabbit bladder.

## Methods

### Tissues

Male New Zealand white rabbits (Burleson Enterprises, Inc., 2.3 – 2.7 kg) were euthanized by halothane inhalation according to an IACUC approved protocol. The urinary bladder was isolated at 4°C in a bicarbonate-buffered Krebs solution containing (in mM) 118.0 NaCl, 4.75 KCl, 24.80 NaHCO_3_, 1.18 KH_2_PO_4_, 1.27 CaCl_2_, 1.18 MgSO_4_·7H_2_O, and 10.0 D-glucose saturated with 95% O_2 _and 5% CO_2_. An incision was made from the bladder neck up to the dome following either the dorsal or ventral vasculature. The bladder was pinned out with the mucosa facing down. This protocol caused ridges to form from which strips were dissected from the abluminal surface. Histological examination showed that the cells were aligned in the longitudinal axis of the preparation in which length and force were measured (not illustrated). The bladder strips were tied to the two posts on the apparatus using silk sutures; one post to a micrometer to change length, and the other to a FT0.3 Grass force transducer. The length was incrementally increased until a constant force of 1 g was maintained, approximating L_o_. The preparations responded with sustained contractions when exposed to 3 μM carbachol. K^+^-depolarization elicited transient contractions diagnostic of a phasic smooth muscle [9]. Tissues exhibiting spontaneous oscillatory activity were excluded from the analysis. A total of 45 tissues were included in the analysis.

### MRLC and HSP20 phosphorylation

Bladder strips treated pharmacologically and then frozen in 20 ml of acetone cooled with 20 ml crushed dry ice. They were then slowly (2.5 hours) thawed to room temperature to dehydrate the tissues, air dried and weighed. The dry samples were homogenized in ground glass tissue homogenizers on ice in 1% (w/v) sodium dodecyl sulfate (SDS), 10% (v/v) glycerol, 0.1% pefabloc (a protease inhibitor), 0.1 % microcystin, and 30 mM dithiothreitol (0.22 ml/mg tissue dry weight), and then centrifuged at 14,000 × g for 10 min. Trichorloacetic acid was not included since it did not alter MRLC phosphorylation estimates. Serial dilutions (1/2, 1/4, 1/8, 1/16 and 1/32) of homogenates in homogenization buffer were loaded onto 12% acrylamide/glycerol-urea slab gels for isoelectric focusing at 250 volts overnight on a pH 4.0–6.5 gradient for MRLC [10] and a pH 4.5–8.0 gradient for HSP20 [6]. Gels were focused at 250 V constant voltage for 12 h at 8°C. Separated proteins were transferred to a nitrocellulose membrane by electroblotting in Towbin's transfer buffer (25 mM Tris, 192 mM glycine, 20% methanol, 0.1% SDS) at 200 mA constant current for 2 h at 8°C. The membranes were first washed in a 0.1% Tris-buffered saline-Tween solution (TBST: 10 mM Tris, 0.05% NaCl, 0.1% Tween-20). The membranes were then blocked overnight in TBST containing 1% bovine serum albumin and 0.01% sodium azide. After rinsing in TBST, the membranes were incubated in either 1:2000 anti-MRLC antibody (20 kD MRLC from Sigma) or 1:1000 rabbit anti-HSP20 (made by the authors) antibody for 1 h. After rinsing in TBST, the membranes were incubated with a horseradish peroxidase conjugate secondary (1:15000) for 1 h. After rinsing twice with TBST and once with TBS (TBST without Tween-20), antibodies were detected with enhanced chemiluminescence was scanned using a Molecular Dynamics laser densitometer and analyzed by NIH Image software. The relative protein content was estimated assuming that antibody binding was the same for phospho- and dephospho-MRLC and corrections made for offset and saturation errors as described [10].

## Results

Carbachol (0.3 μM) alone induced a sustained contraction that measured 56 ± 9 % of a maximal (3 μM) carbachol contraction. Therefore, 0.3 μM carbachol was used to test relaxing agents. Preliminary experiments showed that 0.3 μM carbachol stimulated rabbit bladder did not relax with 100 μM nitroprusside (data not shown). Carbachol (0.3 μM) stimulated rabbit bladder relaxed when treated with 8-bromo-cGMP (a cell permeant cGMP analog – force was 30 ± 4 % of a 3 μM carbachol contraction), forskolin (a non receptor activator of adenyl cyclase – force was 12 ± 5 %), or isoprenaline (a non receptor activator of adenyl cyclase – force was 18 ± 8 %).

**Figure 1:**
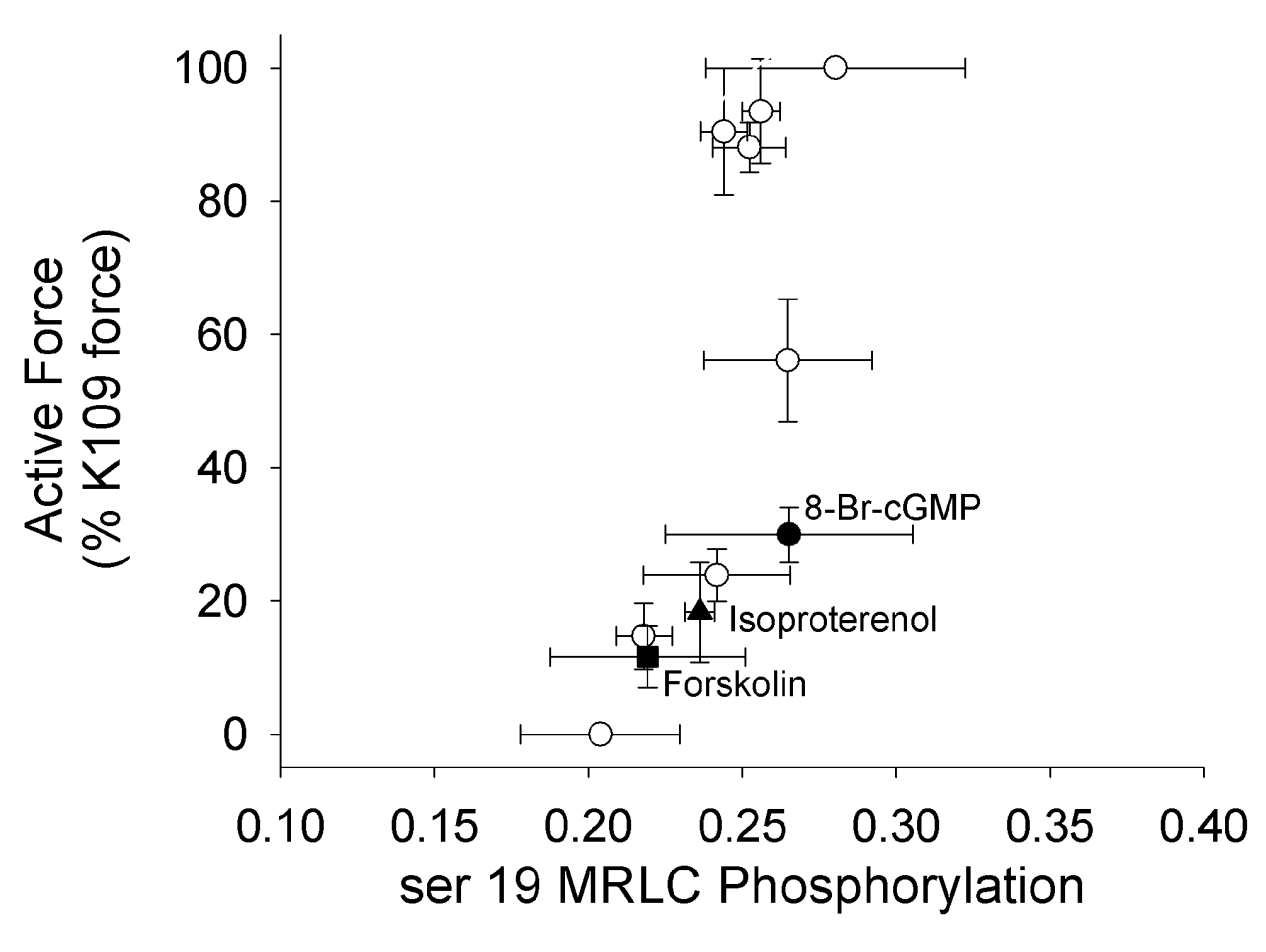
The steady-state relationship between active force and percent MRLC phosphorylation in the rabbit urinary bladder detrusor strips. Force is expressed as a percent of the initial response to 3 μM carbachol. The open circles show results from tissues were stimulated by varying doses of carbachol (0.01, 0.03, 0.1, 0.3, 1.0, 3.0, 10, 30, and 100 μM) until steady state was achieved (the data with carbachol alone previously published [11]). The symbol at 56% of force represents tissues stimulated with 0.3 μM carbachol. The filled symbols show results from tissues were stimulated 0.3 μM carbachol until steady state was achieved followed by addition of 300 μM 8-bromo-cGMP (filled circle), 10 μM forskolin (filled square), or 0.3 μM isoprenaline (filled triangle) until steady state was achieved.

The open circles in Fig. 1 shows the steep dependence of force on MRLC phosphorylation when rabbit urinary bladder was activated with 0.01–100 μM carbachol (these data were previously published [11]). The filled symbols in Fig. 1 show the dependence of force on MRLC phosphorylation when 0.3 μM carbachol stimulated rabbit bladder were relaxed with 8-bromo-cGMP (filled circle), forskolin (square), or isoprenaline (triangle). These three treatments induced a dependence of force on MRLC phosphorylation that did not differ from that observed with carbachol alone (open circles), suggesting the relaxation occurred by deactivation. If there had been force suppression, there would have been a rightward shift in the dependence of force on MRLC phosphorylation.

**Figure 2:**
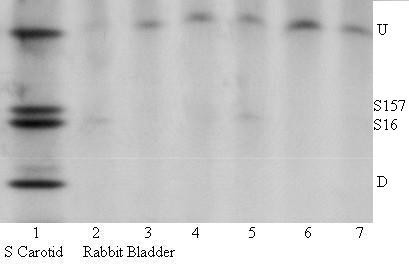
Representative HSP20 immunoblot of homogenates from a swine carotid tissue (lane 1, 15 μl loaded) and six different rabbit bladder tissues (lanes 2–7, 30 μl loaded). The bands have been identified in swine carotid as unphosphorylated (labeled U), monophosphorylated at serine 157 (labeled S157), monophosphorylated at serine 16 (labeled S16), diphosphorylated at serine 16 and 157 (labeled D), There was significantly less HSP20 immunostaining in the bladder compared to swine carotid, suggesting that the HSP20 in bladder could come from contaminating vasculature.

HSP20 immunostaining was low in rabbit bladder (Fig. 2, lanes 2–7) compared to swine carotid (Fig. 2, left lane 1). Overall, there was 70.5 fold less HSP20 immunostaining in the bladder homogenates compared to swine carotid homogenates. Since this comparison was normalized on tissue dry weight, we also compared MRLC immunostaining from the same samples. There was 2.9 fold more MRLC immunostaining in the bladder homogenates compared to swine carotid homogenates (data not shown). When HSP20 immunostaining was normalized by MRLC immunostaining, there was 204 fold less HSP20 immunostaining in the bladder compared to the swine carotid immunostaining.

## Discussion

These data suggest that rabbit bladder does not express significant levels of HSP20 (Fig. 2) and does not exhibit force suppression (Fig. 1). This result is consistent with the hypothesis that HSP20 is the mediator of force suppression [7]. Our results are reminiscent of data showing that umbilical vein does not express significant levels of HSP20 and does not relax to NO donors [8].

We think it most likely that the low level of HSP20 immunostaining is caused by HSP20 present in the vascular smooth muscle of the bladder vasculature. However, we cannot rule out a low level of HSP20 expression in bladder smooth muscle. We assumed that carotid and bladder have similar cellular MRLC concentration (13) so that MRLC immunostaining could be used to normalize HSP20 immunostaining. The accuracy of this assumption is not that crucial given the relatively small amount of HSP20 immunostaining in the bladder (approximately two hundred-fold less).

We confirmed that the rabbit bladder does not relax to NO donors. However, there was a relaxation to 8-bromo-cGMP, a cell permeant cGMP analog, suggesting that the lack of response to NO donors resides in the generation of cGMP, rather than a defect in the response to cGMP. Non-receptor activators of adenyl cyclase (forskolin and isoprenaline) also induced relaxation, suggesting that rabbit bladder can relax to increases in cAMP. Importantly, these relaxations cGMP and cAMP mediated relaxations were not associated with force suppression (which would have produced a rightward shift in the dependence of force on MRLC phosphorylation). This is consistent with the hypothesis that HSP20 is involved in force suppression. The relaxations in response to cGMP analogs and agents that increase [cAMP] suggest that the relaxation is caused by deactivation mechanisms such as mechanisms that reduce [Ca^2+^]_i _or possibly increase myosin phosphatase activity [12,13].

## Conclusion

These results suggest that some phasic smooth muscles, such as rabbit bladder, do not exhibit force suppression. Force suppression was found in corpus cavernosum [4,5], a NO responsive phasic smooth muscle. Unlike the human umbilical vein study [8], we report that the lack of HSP20 is not only associated with a lack in relaxation, but also a lack of force suppression.

## Competing interests

The author(s) declare that they have no competing interests.

## Authors' contributions

TB and JW performed the studies. RM and CR conceived the study. TB and CR drafted the study.
